# Robo4 Plays a Role in Bone Marrow Homing and Mobilization, but Is Not Essential in the Long-Term Repopulating Capacity of Hematopoietic Stem Cells

**DOI:** 10.1371/journal.pone.0050849

**Published:** 2012-11-30

**Authors:** Yuko Goto-Koshino, Yumi Fukuchi, Fumi Shibata, Daichi Abe, Kana Kuroda, Shinichiro Okamoto, Toshio Kitamura, Hideaki Nakajima

**Affiliations:** 1 Division of Cellular Therapy, Advanced Clinical Research Center, The Institute of Medical Science, The University of Tokyo, Tokyo, Japan; 2 Division of Hematology, Department of Internal Medicine, Keio University School of Medicine, Tokyo, Japan; Emory University, United States of America

## Abstract

Roundabout (Robo) family proteins are immunoglobulin-type surface receptors critical for cellular migration and pathway finding of neuronal axons. We have previously shown that Robo4 was specifically expressed in hematopoietic stem and progenitor cells and its high expression correlated with long-term repopulating (LTR) capacity. To reveal the physiological role of Robo4 in hematopoiesis, we examined the effects of Robo4 disruption on the function of hematopoietic stem cells (HSCs) and progenitors. In Robo4-deficient mice, basic hematological parameters including complete blood cell count and differentiation profile were not affected. In contrast to the previous report, HSC/hematopoietic progenitor (HPC) frequencies in the bone marrow (BM) were perfectly normal in Robo4^−/−^ mice. Moreover, Robo4^−/−^ HSCs were equally competitive as wild-type HSCs in transplantation assays and had normal long-term repopulating (LTR) capacity. Of note, the initial engraftment at 4-weeks after transplantation was slightly impaired by Robo4 ablation, suggesting a marginal defect in BM homing of Robo4^−/−^ HSCs. In fact, homing efficiencies of HSCs/HPCs to the BM was significantly impaired in Robo4-deficient mice. On the other hand, granulocyte-colony stimulating factor-induced peripheral mobilization of HSCs was also impaired by Robo4 disruption. Lastly, marrow recovery from myelosuppressive stress was equally efficient in WT- and Robo4-mutant mice. These results clearly indicate that Robo4 plays a role in HSC trafficking such as BM homing and peripheral mobilization, but is not essential in the LTR and self-renewal capacity of HSCs.

## Introduction

Hematopoietic stem cells (HSCs) are a rare population of cells that can support life-long hematopoiesis, that are characterized by the unique capacity to self-renew and differentiate into all blood cell lineages. HSCs reside in the specific microenvironment known as the niche in the adult bone marrow (BM). The niche is thought to be located on the surface of trabecular bones or near the marrow sinusoids, and osteoblasts or endothelial cells serve as the niche for HSCs [1]–[2]. Side population (SP) phenotype defined as the activity of Hoechst 33342 dye efflux is one of the hallmarks of quiescent HSCs in the BM niche [3]–[4], and it has been shown that many of the quiescent HSCs reside in c-Kit^+^Sca-1^+^Lineage^-^ (KSL)-SP population. Interestingly, quiescent HSCs move from SP to a main population (MP) of non-SP cells, which represents transient amplifying multipotent progenitors, when they are recruited into the cell cycle upon myelosuppressive stimuli such as 5-fluorouracil (5-FU) treatment [5].

Roundabout (Robo) family proteins are immunoglobulin-type receptors that play critical roles in cellular migration and pathway findings of neurons [6]–[7]. Robo receptors elicit intracellular signals to modulate cell motility by binding to the cognate ligand, Slit proteins [8]. Although many of the Robo-Slit functions have been analyzed in neuronal systems, some reports described their critical roles in lymphocyte migration [9] and tumor angiogenesis [10]. In a search for molecules specifically expressed in HSCs, we found that Robo4, the forth member of Robo family, is highly expressed in HSCs and suggested its role in the regulation of their side population phenotype [11]. We have also shown that Slit2 is specifically induced in osteoblasts in the BM in response to myelosuppressive stimuli, suggesting that Slit2 may play a role in recruiting quiescent HSCs into the cell cycle.

Analysis of Robo4-deficient mice is necessary in order to elucidate the physiological role of Robo4 in vivo. We herein show that Robo4 is not absolutely required for homeostasis of hematopoietic stem/progenitor cells and mature hematopoietic cells in steady-state settings. Importantly, complete blood cell counts, differentiation profile of peripheral blood and bone marrow cells, and HSC/hematopoietic progenitor (HPC) frequencies were perfectly normal in Robo4^−/−^ mice as compared to wild-type. Moreover, Robo4^−/−^ HSCs were similarly competitive as wild-type HSCs in transplantation assays and could repopulate the marrow for long-term to the same level as wild-type HSCs. On the other hand, BM homing and mobilization of HSCs were significantly impaired in Robo4^−/−^ mice. Some of our data are in clear contrast to the recent report showing decreased HSC frequency and impaired long-term repopulating (LTR) activity of HSCs in Robo4-deficient mice [12]. Discrepancies between these two data call further investigation to elucidate precise roles of Robo4 in HSC functions.

## Materials and Methods

### Mice

C57BL/6 (B6) mice were from Japan CLEA Inc. (Tokyo, Japan), and B6-Ly5.1 mice were from Sankyo Lab Service Co. (Tsukuba, Japan). Robo4-deficient mice were described previously [13]–[14] and were backcrossed to C57BL/6 more than 7 times before performing experiments. All mice were kept under specific pathogen-free conditions. Mice from 8- to 12-weeks old were used in all experiments. All animal experiments were reviewed and approved by the Internal Review Board of the Institute of Medical Science, the University of Tokyo and by the committee of animal use and care of Keio University School of Medicine.

### Antibodies

Anti-mouse c-Kit-APC, Sca-1-FITC, Sca-1-PE-Cy7, Flt3-PE and CD34-FITC antibodies were from BD-Pharmingen. Other lineage-specific antibodies (anti-CD3, CD4, CD8, CD11b, Gr-1, CD41, CD43, B220, Ter119) were purchased from e-Bioscience. Anti-Robo4 antibody was described previously [11].

### Flow Cytometry

BM cells were obtained by flushing out femurs and tibias from 8- to 12-week-old mice with phosphate-buffered saline (PBS). Depletion of lineage-positive cells, staining and FACS sorting of KSL, CD34^−^KSL, CD34^−^Flt3^−^KSL, CD34^+^Flt3^−^KSL, CD34^+^Flt3^+^KSL cells were described previously [15], [16]. Hoechst staining of lineage-depleted cells for the analysis of KSL-SP or KSL-MP was performed as previously described by Goodell et al. [3]. Stained cells were analyzed by FACS Aria or FACS Vantage (Beckton Dickinson).

### Colony-forming Cell Assay

For the colony-forming cell assay, BM mononuclear cells were deposited into MethoCult GF M3434 (Stem Cell Technologies), mixed, and were plated onto Petri-dish in triplicate. Number and type of colonies were assessed at day 7 of culture.

### Mobilization of Hematopoietic Progenitors to Peripheral Blood

Wild-type or Robo4-mutant mice were treated with granulocyte-colony stimulating factor (G-CSF) (250 µg/kg, s.c.) for 5 days. Cells were harvested from BM or peripheral blood (PB) at 6 hours after the last dose of G-CSF, and the numbers of hematopoietic progenitors (CFU-GM) were assessed by colony assays.

### Bone Marrow Transplantation

Bone marrow transplantation was performed as previously described [17] using B6-Ly5.1 mice (Sankyo Lab Service Co.) as recipients and competitors. For competitive repopulation assay, 100 CD34^−^KSL cells for each group were sorted from BM mononuclear cells of WT or Robo4-mutant mice (Ly5.2) and transplanted with 2×10^5^ competitor cells (Ly5.1) into lethally irradiated recipient mice (Ly5.1).

### In vivo Homing Assay

1×10^7^ bone marrow mononuclear cells (BMMNCs) were harvested from wild-type or Robo4-mutant mice and were transplanted into lethally irradiated (950 rads) recipients. 48 hours after the transplant, BM cells were taken from the recipient femurs and were subjected to colony assays. Alternatively, 1×10^4^ c-Kit^+^Lin^−^ cells were sorted from WT- or Robo4^−/−^ BM cells and were labeled with CSFE for 5 minutes at room temperature. Labeled cells were then transplanted into lethally irradiated recipient mice. 16 hours after transplantation, BM cells were harvested from recipient mice and analyzed for CSFE-positive cells by flow cytometry.

### RT-PCR

PolyA^+^ mRNA was extracted from BM cells using a Micro-FastTrack 2.0 Kit (Invitrogen). Synthesis of complementary DNA and standard RT-PCR was performed as previously described [15]. Quantitative RT-PCR was performed as described previously using ABI StepOne Plus real-time PCR system (Applied Biosystems) [15]. cDNA quantity was normalized by the level of GAPDH as an endogenous control. Primer sequences were previously described [12].

### Statistical Analysis

Statistical analyses were performed using unpaired Student’s t-test. P-values less than 0.05 were regarded as statistically significant.

## Results

### Disruption of Robo4 does not Affect the Frequency of Hematopoietic Stem and Progenitor Cells in the BM

To investigate physiological roles of Robo4 in hematopoiesis, we analyzed Robo4-deficient mice to see if there were any defects in the function of HSCs/HPCs. As previously reported, Robo4-deficient mice exhibited complete loss of the mRNA and the protein, and importantly, the expression of other Robo-family members (Robo1, Robo2, Robo3) was not affected by Robo4 disruption in non-hematopoietic tissues and spleen [13]. We have also confirmed the loss of Robo4 in Robo4^−/−^ HSC/HPC fraction by RT-PCR and flow cytometry (Figure S1A, B and C). Notably, compensatory up-regulation of other Robo family members was not observed in Robo4^−/−^ KSL cells (Figure S1D). It was reported that CXCR4 was up-regulated in Robo4^−/−^ HSCs [12] and we confirmed this data by quantitative RT-PCR (Figure S1E).

Next we examined basic hematological parameters of Robo4-deficient mice. The results showed that the complete blood cell counts were not statistically different between WT and Robo4-deficient animals (Table 1). In addition, flow cytometric profiles of the BM, spleen and thymus were completely normal in Robo4^−/−^ mice as compared to WT, indicating that Robo4 is not required for the differentiation of myeloid, erythroid/megakaryocytic, and lymphoid lineages (Figure S2, S3, S4). Colony assays revealed that the numbers of committed progenitors such as colony forming unit-granulocyte macrophage (CFU-GM), burst forming unit-erythroid (BFU-E) and erythroid-mix (E-mix) in the BM or spleen were not significantly different between WT and Robo4-mutant animals (Figure S5).

**Table 1:** Complete blood cell count of Robo4-mutant mice.

	+/+	−/−
WBC (×10^2^/µl)	131.5±22.1	126.4±17.6
Hb (g/dl)	16.6±1.2	17.3±2.1
PLT (×10^4^/µl)	60.3±21.0	59.3±23.8

Data are mean +/− S.D. (n = 5 for each genotype).

**Figure 1:**
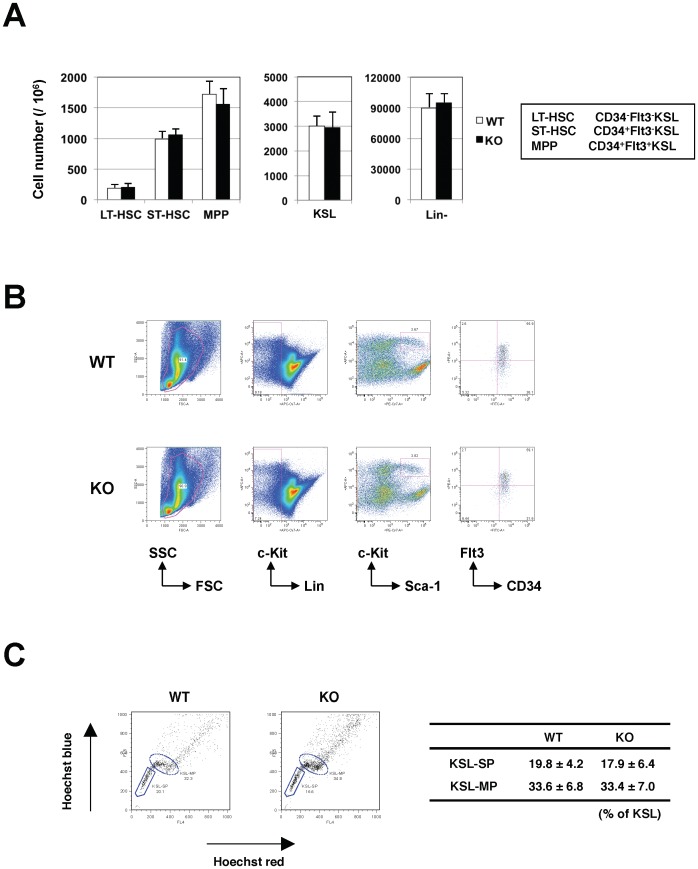
Frequency of hematopoietic stem and progenitors in wild-type and Robo4-mutant mice. (A) Actual numbers of long-term (LT)-HSCs (CD34^−^Flt3^−^KSL), short-tem (ST)-HSCs (CD34^+^Flt3^−^KSL), multipotent progenitors (MPPs; CD34^+^Flt3^+^KSL), KSL and lineage negative (Lin^−^) cells in WT or Robo4-deficient mice (mean+/−S.D., n = 5). (B) Representative FACS figures for LT-HSC, ST-HSC and MPP. (C) Frequencies of KSL-SP and KSL-MP cells in WT or Robo4-mutant mice (mean+/−S.D., n = 3). Representative FACS figures are shown on the left.

We have previously reported that Robo4 is highly expressed on HSCs [11], and therefore speculated that disruption of Robo4 might have impacts on their physiological function and homeostasis. Unexpectedly however, the frequencies of long-term (LT)-HSCs (CD34^−^Flt3^−^KSL), short-term (ST)-HSCs (CD34^+^Flt3^−^KSL) and multipotent progenitors (MPPs) (CD34^+^Flt3^+^KSL) as well as KSL and Lin^−^ cells were not significantly different between WT and Robo4-deficient animals (Figure 1A and B). In addition, the percentages of SP or MP fraction in KSL cells were normal in Robo4-mutant animals as compared to WT (Figure 1C).

These results clearly demonstrate that disruption of Robo4 has no or little, if any, impact on hematopoietic differentiation and the frequencies of LT-HSCs, ST-HSCs and MPPs as well as committed progenitors in the BM.

### Disruption of Robo4 does not Affect the Long Term Repopulating Capacity of HSCs

**Figure 2:**
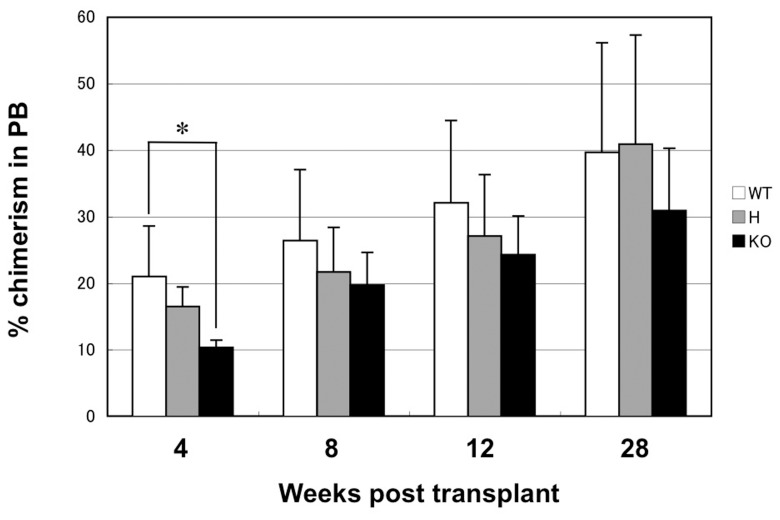
Long-term repopulation assay of HSCs. One hundred CD34^−^KSL cells from WT or Robo4-mutant mice (Ly5.2) were transplanted into lethally irradiated recipients (Ly5.1) with 2×10^5^ competitors. Percentage of donor chimerism (Ly5.2) in peripheral blood was examined at the indicated time points. Data are shown as mean +/− S.D. (n = 6 for WT and H, n = 8 for KO). *p<0.05. WT; wild-type, H; heterozygous, KO; knockout.

We next investigated whether the LTR capacity was impaired in Robo4^−/−^ HSCs. We sorted one hundred CD34^−^KSL cells from the BM of WT or Robo4-mutant mice by flow cytometry, and transplanted them into lethally irradiated recipients with 2×10^5^ competitor cells. As shown in Figure 2, Robo4^−/−^ CD34^−^KSL cells showed significantly reduced engraftment at 4-weeks after transplantation as compared to WT. However, donor chimerism in PB at longer time points (8-, 12- and 28-weeks after transplantation) was not statistically different between WT, Robo4^+/−^ and Robo4^−/−^ cells. Of note, donor cells of each genotype showed efficient multi-lineage differentiation including myeloid, B and T lineages, again confirming that the differentiation potential was not affected by the loss of Robo4 (data not shown). These data clearly indicate that the disruption of Robo4 may partially impair short-term repopulating potential of HSCs, but it scarcely affects their overall LTR capacity.

### A Role of Robo4 in Homing of Hematopoietic Stem/Progenitor Cells to the Bone Marrow

**Figure 3:**
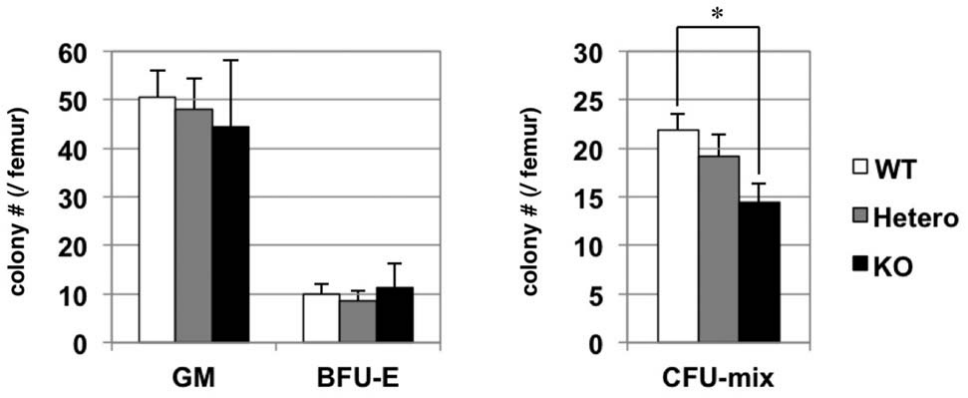
Bone marrow homing capacity of hematopoietic progenitors from Robo4-mutant mice. 1×10^7^ BM mononuclear cells (BMMNCs) were harvested from WT or Robo4-mutant mice and were transplanted into lethally irradiated recipients. 24 hours after the transplant, BM cells were harvested from the recipient femurs and were subjected to colony assays. GM; colony forming unit (CFU)-granulocyte/macrophage, BFU-E; burst forming unit-erythroid, CFU-mix; colony forming unit-mix. Data are mean +/− S.D. (n = 6). *p<0.05. BM cells taken from the mice that had not been transplanted (negative control) did not generate colonies (data not shown).

Since Slit-Robo signaling plays a critical role in cellular migration, we speculated that Robo4 might be important for regulating homing of HSCs/HPCs to the BM. This hypothesis was strengthened by our previous observation that Slit2, a natural ligand for Robo4, was expressed exclusively in osteoblasts in the BM, a critical component of HSC niche. In order to test this hypothesis, we performed BM homing assay using the BM cells from WT and Robo4-mutant mice. In this assay, 1×10^7^ BM mononuclear cells were transplanted into lethally irradiated recipients and the number of HSCs/HPCs homed in the recipient’s marrow 48-hours after the transplant was examined by colony assay (Figure 3). The advantage of this assay was the frequencies of HSCs/HPCs and committed progenitors homed in the BM can be assessed simultaneously by the numbers of colony forming unit (CFU)-mix and CFU-granulocyte/macrophage (GM)/burst forming unit-erythroid (BFU-E), respectively. Interestingly, the homing frequency of Robo4^−/−^ HSCs/HPCs as assessed by CFU-mix was significantly lower than WT ones, while the number of committed progenitors such as CFU-GM or BFU-E was not significantly different between WT and Robo4-mutant cells. We have also analyzed the homing efficiency of Robo4^−/−^ HSCs/HPCs by labeling cells with CSFE-dye. Again, this revealed that Robo4 disruption reduced BM homing efficiency of HSCs/HPCs, even more drastically at shorter time point (16 hours) after transplantation (Figure S6).

Taken together, these data suggest that Robo4 plays a role in the BM homing of HSCs/HPCs, but is dispensable for that of committed progenitors.

### A Role of Robo4 in Peripheral Mobilization of Hematopoietic Progenitors from the BM

**Figure 4:**
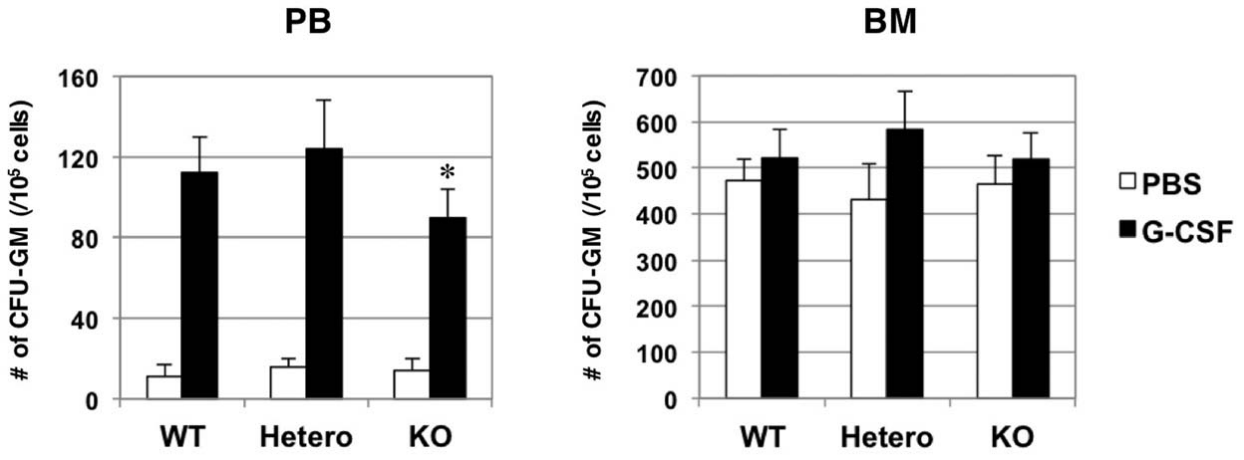
Mobilization of hematopoietic progenitors by G-CSF treatment. WT or Robo4-mutant mice were treated with G-CSF for 5days. BM and peripheral blood (PB) were harvested at 6 hours after the last dose of G-CSF, and the numbers of CFU-GM per 1×10^5^ BM or PB cells were assessed by colony assays. Data are mean +/− S.D. (n = 6). *p<0.05.

Given a role for Robo4 in the homing of HSCs/HPCs into the BM, we speculated that it may also play a role in the complementary process, HSC/HPC mobilization into the PB. To mobilize HSCs/HPCs from the BM, G-CSF was subcutaneously administered to WT and Robo4-mutant mice, and the number of mobilized progenitors in peripheral circulation was assessed by colony assays. As shown in Figure 4, treating mice with G-CSF for 5-days increased the number of progenitors in PB of WT and Robo4-mutant mice. Interestingly, however, the number of progenitors mobilized to PB was slightly, but significantly lower in Robo4^−/−^ animals as compared to WT. It is of note that the number of progenitors in the BM after the G-CSF treatment was not significantly different between WT and Robo4-mutant mice.

These data suggest that Robo4 may be involved in, but is not absolutely essential for the peripheral mobilization of HSCs/HPCs from the BM.

### A Role of Robo4 in the Emergency Hematopoiesis

**Figure 5:**
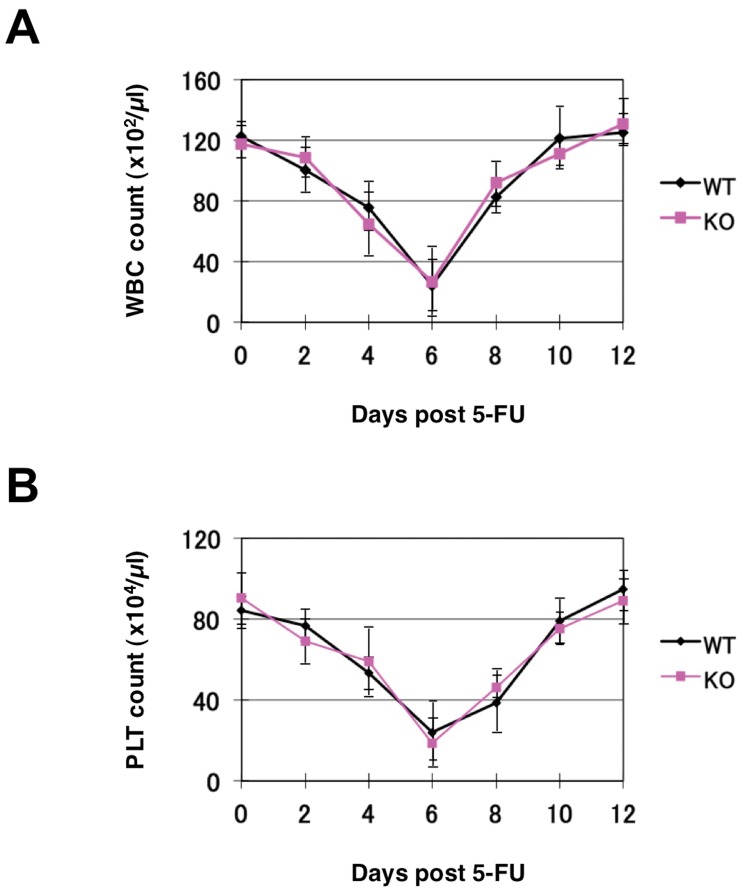
BM recoveries after myelosuppression. WT or Robo4^−/−^ mice were treated with intraperitoneal injection of 5-FU (150 mg/kg). WBC (A) and platelet (PLT) (B) count was monitored at the indicated time points. Data are presented as mean+/−S.D. (n = 5).

The data so far indicate that Robo4 is not required for steady-state hematopoiesis, but may play a role in short-term BM repopulation, homing, and peripheral mobilization of HSCs/HPCs. Another aspect of hematopoietic regulation can be tested in the emergency settings, such as recovery from BM suppression. To assess emergency hematopoiesis, we transiently suppressed hematopoiesis by treating mice with 5-fluorouracil (5-FU), and monitored the recovery of complete blood cell counts. As shown in Figure 5, suppression of white blood cell and platelet counts and recovery from the nadir was not significantly different between WT and Robo4^−/−^ mice at all time points studied. These data suggest that Robo4 is not required for emergency hematopoiesis in response to myelosuppression.

## Discussion

We have previously shown that Robo4 was specifically expressed in murine HSCs and immature progenitor cell fraction, but not in lineage positive cells or differentiated progenitors [11]. We also demonstrated that Slit2 is specifically expressed in osteoblasts and its expression is induced in response to myelosuppressive stress. Furthermore, overexpression of Robo4 or Slit2 in HSCs resulted in their decreased residence in KSL-SP fraction. These results suggested that Robo4/Slit2 signaling plays a role in HSC homeostasis in osteoblastic niche in the BM. This notion is further supported by the observation that Slit/Robo signaling inhibits the function of N-cadherin, a critical component of HSC-osteoblast interaction in the BM niche, in mammalian cells [18].

Based on these findings, we set out to explore physiological function of Robo4 using gene-deficient animals. Unexpectedly, thorough analysis of Robo4^−/−^ mice revealed no obvious difference in the frequencies of HSCs, HPCs or mature hematopoietic cell fractions as compared to the WT. In addition, there was no apparent defect in emergency hematopoiesis in response to myelosuppression. Interestingly, however, we observed slightly decreased BM homing of Robo4^−/−^ HSCs/HPCs as compared to WT, while that of committed progenitors such as CFU-GM or BFU-E were not affected by Robo4 ablation. Furthermore, peripheral mobilization of HSCs/HPCs was slightly, but significantly impaired in Robo4-deficient mice. These data clearly suggest that Robo4 plays a role in the BM trafficking of HSCs/HPCs. The reason for unaffected HSC/HPC frequencies in the Robo4^−/−^ BM despite their defective trafficking is not clear at present. One reason could be that the defects of HSC/HPC trafficking in Robo4-deficient mice are only marginal, which could easily be compensated by other pathways regulating BM trafficking. Another possible reason is that impairments in both homing and mobilization may cancel each other so that net HSC/HPC trafficking into and out of the BM stayed constant. Smith-Berdan et al. have recently reported a critical role of Robo4 in HSC localization to BM niches by examining the same knockout strain as ours [12]. In contrast to our data, they showed that HSC frequency in the BM was decreased in Robo4-deficient mice. They ascribed decreased HSC frequency in the Robo4^−/−^ BM to the defective BM lodgment of Robo4^−/−^ HSCs, although they also observed impaired HSC mobilization in Robo4^−/−^ mice. One reason for the discrepancy between their study and ours might be the genetic background of Robo4-mutant mice, which was the mixed chimera of C57BL/6 and 129/Sv stains [13]–[14]. We backcrossed the strain onto C57BL/6 more than 7 times before experiments, while Smith-Berdan et al. did not precisely mention the background of Robo4-mutant mice. Irrespective, this issue definitely needs further investigation.

One important finding of the current study is that the LTR-capacity was not impaired in Robo4^−/−^ HSCs. While the initial engraftment of Robo4^−/−^ HSCs at 4-weeks after transplantation was slightly decreased, which was consistent with the slight defect in their BM homing, the fact that Robo4^−/−^ HSCs/HPCs can repopulate the marrow for long-term as efficiently as WT ones clearly suggests that Robo4 is not absolutely essential for the long-term BM repopulation of HSCs. Moreover, percentage of donor chimerism in PB increased over time in the recipients of Robo4^−/−^ HSCs similarly as those of WT HSCs, suggesting that self-renewal capacity of Robo4^−/−^ HSCs was not impaired as well. Again, these data are in sharp contrast to the recent report showing a defective LTR capacity of Robo4^−/−^ HSCs [12]. They showed that the percentage of PB chimerism by Robo4^−/−^ HSCs was well below (approximately one-fifth of) the one by WT HSCs, although it was maintained at the same level at least until 16-weeks post-transplant. The reason for the discrepancies between their study and ours is not clear at present, and further studies will definitely be required to elucidate precise role of Robo4 in maintaining frequency and LTR capacity of HSCs.

In summary, our data clearly show that Robo4 is not required for maintaining HSC frequency in the BM and LTR capacity of HSCs. Further studies will be required to clarify the precise role of Robo4 in HSC homeostasis and functions.

## 

## Supporting Information

Figure S1 Expression of Robo1, Robo2, Robo3, Robo4 and CXCR4 in Robo4-mutant mice. (A) Genotyping of WT, Robo4^+/−^ and Robo4^−/−^ mice by genomic PCR. (B) Expression of Robo4 in WT, Robo4^+/−^ and Robo4^−/−^ mice by RT- PCR. BM KSL cells were sorted and subjected to the analysis. NC; negative control. (C) Expression of Robo4 by flow cytometry. Indicated fractions of WT or Robo4^−/−^ BM cells were analyzed for the expression Robo4. (D) Expression of Robo1, Robo2 and Robo3 in KSL cells. KSL cells were sorted from Robo4^+/+^ or Robo4^−/−^ mice and subjected to RT-PCR analysis as described in Materials and Methods. PCR was run for 45 cycles. P; positive control (brain), N; negative control. (E) Expression of CXCR4 in KSL cells. KSL cells sorted from Robo4^+/+^ or Robo4^−/−^ BM cells were subjected to RNA extraction and quantitative RT-PCR as described in Materials and Methods. The level of expression is shown relative to the one in Robo4^+/+^ cells (mean+/−S.D., n = 3). Difference was statistically significant by Student’s t-test (p<0.05).
(TIF)

Figure S2 Flow cytometric analysis of bone marrow cells from WT and Robo4^−/−^ mice. BM mononuclear cells were stained and analyzed as described in Materials and Methods using the antibodies shown on the right.
(TIF)

Figure S3 Flow cytometric analysis of spleen cells from WT and Robo4^−/−^ mice. Spleen cells were stained and analyzed as described in Materials and Methods using the antibodies shown on the right.
(TIF)

Figure S4 Flow cytometric analysis of thymocytes from WT and Robo4^−/−^ mice. Thymocytes were stained and analyzed as described in Materials and Methods using the antibodies shown on the right.
(TIF)

Figure S5 Colony assays of bone marrow or spleen cells from WT and Robo4-mutant mice. Colony assays were performed as described in Materials and Methods. Data are shown as number of colonies per 1×10^5^ cells plated (mean+/−S.D., n = 3). GM; colony forming unit (CFU)-granulocyte/macrophage, BFU-E; burst forming unit-erythroid, E-mix; erythroid-mix. KO; Robo4^−/−^, Hetero; Robo4^+/−^.
(TIF)

Figure S6 Bone marrow homing assay of Robo4-mutant cells. Immature hematopoietic cells (c-Kit^+^Lin^-^ cells) were sorted from WT or Robo4^−/−^ BM cells and were labeled with CSFE. Labeled cells were then transplanted into lethally irradiated recipient mice. 16 hours after transplantation, BM cells were harvested from recipient mice and analyzed for CSFE-positive cells by flow cytometry. The data are mean+/−S.D. (n = 3). Difference was statistically significant by Student’s t-test (p<0.05).
(TIF)

## References

[1] Zhang J, Niu C, Ye L, Huang H, He X, et al (2003) Identification of the haematopoietic stem cell niche and control of the niche size. Nature 425: 836–841. 14574412 10.1038/nature02041

[2] Calvi LM, Adams GB, Weibrecht KW, Weber JM, Olson DP, et al (2003) Osteoblastic cells regulate the haematopoietic stem cell niche. Nature 425: 841–846. 14574413 10.1038/nature02040

[3] Goodell MA, Brose K, Paradis G, Conner AS, Mulligan RC (1996) Isolation and functional properties of murine hematopoietic stem cells that are replicating in vivo. J Exp Med 183: 1797–1806. 8666936 10.1084/jem.183.4.1797 PMC2192511

[4] Goodell MA, Rosenzweig M, Kim H, Marks DF, DeMaria M, et al (1997) Dye efflux studies suggest that hematopoietic stem cells expressing low or undetectable levels of CD34 antigen exist in multiple species. Nat Med 3: 1337–1345. 9396603 10.1038/nm1297-1337

[5] Arai F, Hirao A, Ohmura M, Sato H, Matsuoka S, et al (2004) Tie2/angiopoietin-1 signaling regulates hematopoietic stem cell quiescence in the bone marrow niche. Cell 118: 149–161. 15260986 10.1016/j.cell.2004.07.004

[6] Wong K, Park HT, Wu JY, Rao Y (2002) Slit proteins: molecular guidance cues for cells ranging from neurons to leukocytes. Curr Opin Genet Dev 12: 583–591. 12200164 10.1016/s0959-437x(02)00343-x

[7] Rajagopalan S, Nicolas E, Vivancos V, Berger J, Dickson BJ (2000) Crossing the midline: roles and regulation of Robo receptors. Neuron 28: 767–777. 11163265 10.1016/s0896-6273(00)00152-5

[8] Brose K, Bland KS, Wang KH, Arnott D, Henzel W, et al (1999) Slit proteins bind Robo receptors and have an evolutionarily conserved role in repulsive axon guidance. Cell 96: 795–806. 10102268 10.1016/s0092-8674(00)80590-5

[9] Wu JY, Feng L, Park HT, Havlioglu N, Wen L, et al (2001) The neuronal repellent Slit inhibits leukocyte chemotaxis induced by chemotactic factors. Nature 410: 948–952. 11309622 10.1038/35073616 PMC2072862

[10] Wang B, Xiao Y, Ding BB, Zhang N, Yuan X, et al (2003) Induction of tumor angiogenesis by Slit-Robo signaling and inhibition of cancer growth by blocking Robo activity. Cancer Cell 4: 19–29. 12892710 10.1016/s1535-6108(03)00164-8

[11] Shibata F, Goto-Koshino Y, Morikawa Y, Komori T, Ito M, et al (2009) Roundabout 4 is expressed on hematopoietic stem cells and potentially involved in the niche-mediated regulation of the side population phenotype. Stem Cells 27: 183–190. 18927479 10.1634/stemcells.2008-0292 PMC2883560

[12] Smith-Berdan S, Nguyen A, Hassanein D, Zimmer M, Ugarte F, et al (2011) Robo4 cooperates with CXCR4 to specify hematopoietic stem cell localization to bone marrow niches. Cell Stem Cell 8: 72–83. 21211783 10.1016/j.stem.2010.11.030 PMC3625377

[13] Jones CA, London NR, Chen H, Park KW, Sauvaget D, et al (2008) Robo4 stabilizes the vascular network by inhibiting pathologic angiogenesis and endothelial hyperpermeability. Nat Med 14: 448–453. 18345009 10.1038/nm1742 PMC2875252

[14] Jones CA, Nishiya N, London NR, Zhu W, Sorensen LK, et al (2009) Slit2-Robo4 signalling promotes vascular stability by blocking Arf6 activity. Nat Cell Biol 11: 1325–1331. 19855388 10.1038/ncb1976 PMC2854659

[15] Nakajima H, Ito M, Smookler DS, Shibata F, Fukuchi Y, et al (2010) TIMP-3 recruits quiescent hematopoietic stem cells into active cell cycle and expands multipotent progenitor pool. Blood 116: 4474–4482. 20798233 10.1182/blood-2010-01-266528

[16] Ema H, Morita Y, Yamazaki S, Matsubara A, Seita J, et al (2006) Adult mouse hematopoietic stem cells: purification and single-cell assays. Nat Protoc 1: 2979–2987. 17406558 10.1038/nprot.2006.447

[17] Nakajima H, Shibata F, Fukuchi Y, Goto-Koshino Y, Ito M, et al (2006) Immune suppressor factor confers stromal cell line with enhanced supporting activity for hematopoietic stem cells. Biochem Biophys Res Commun 340: 35–42. 16343424 10.1016/j.bbrc.2005.11.146

[18] Rhee J, Mahfooz NS, Arregui C, Lilien J, Balsamo J, et al (2002) Activation of the repulsive receptor Roundabout inhibits N-cadherin-mediated cell adhesion. Nat Cell Biol 4: 798–805. 12360290 10.1038/ncb858

