# Supplementary figures and images for: Robo4 Plays a Role in Bone Marrow Homing and Mobilization, but Is Not Essential in the Long-Term Repopulating Capacity of Hematopoietic Stem Cells
Source: PLoS One. 2012 Nov 30;7(11):e50849. doi: 10.1371/journal.pone.0050849 (PMC3511340; doi:10.1371/journal.pone.0050849)

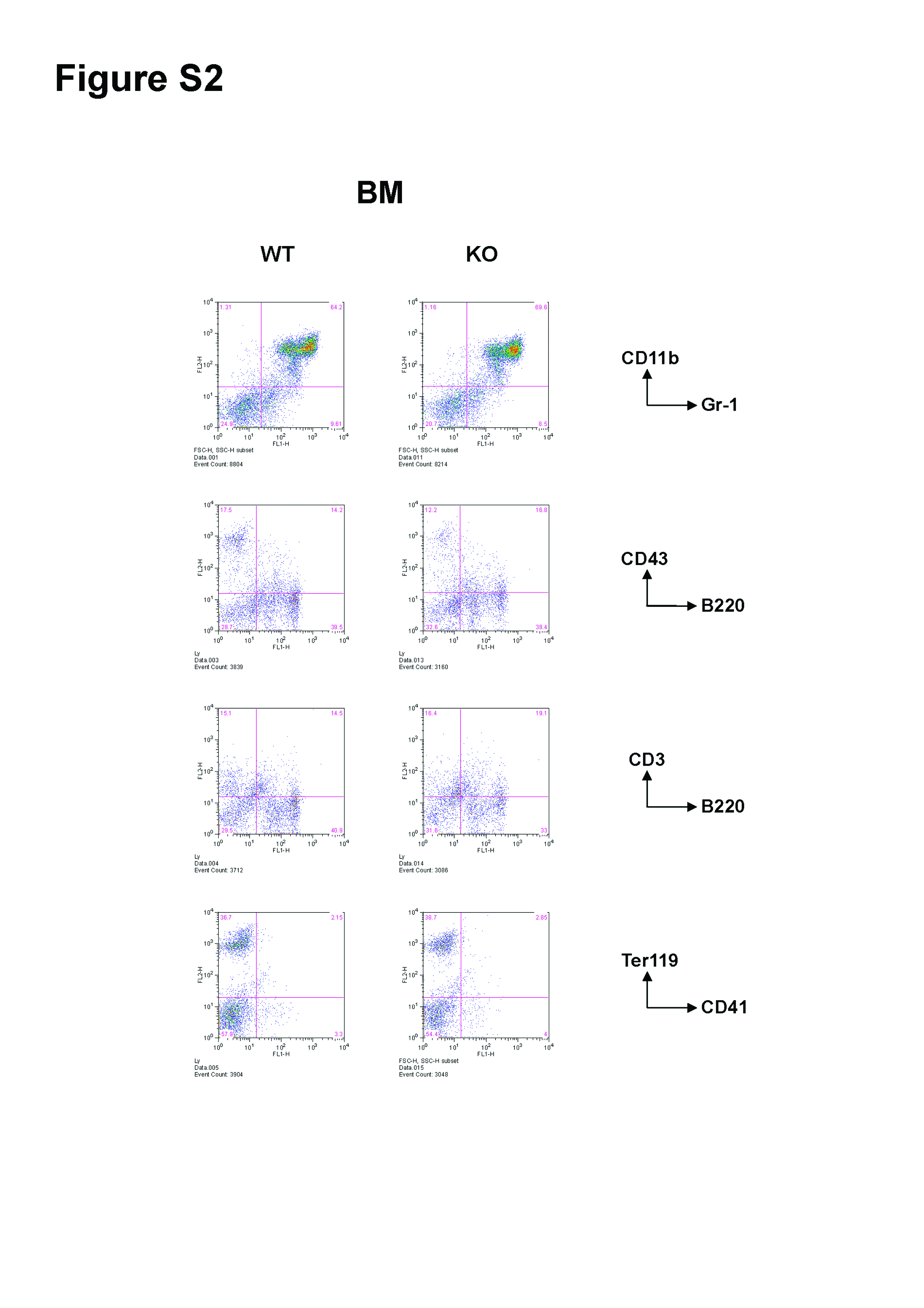

Supplement: Figure S2 — Flow cytometric analysis of bone marrow cells from WT and Robo4−/− mice. BM mononuclear cells were stained and analyzed as described in Materials and Methods using the antibodies shown on the right. (TIF) [file pone.0050849.s002.tif]

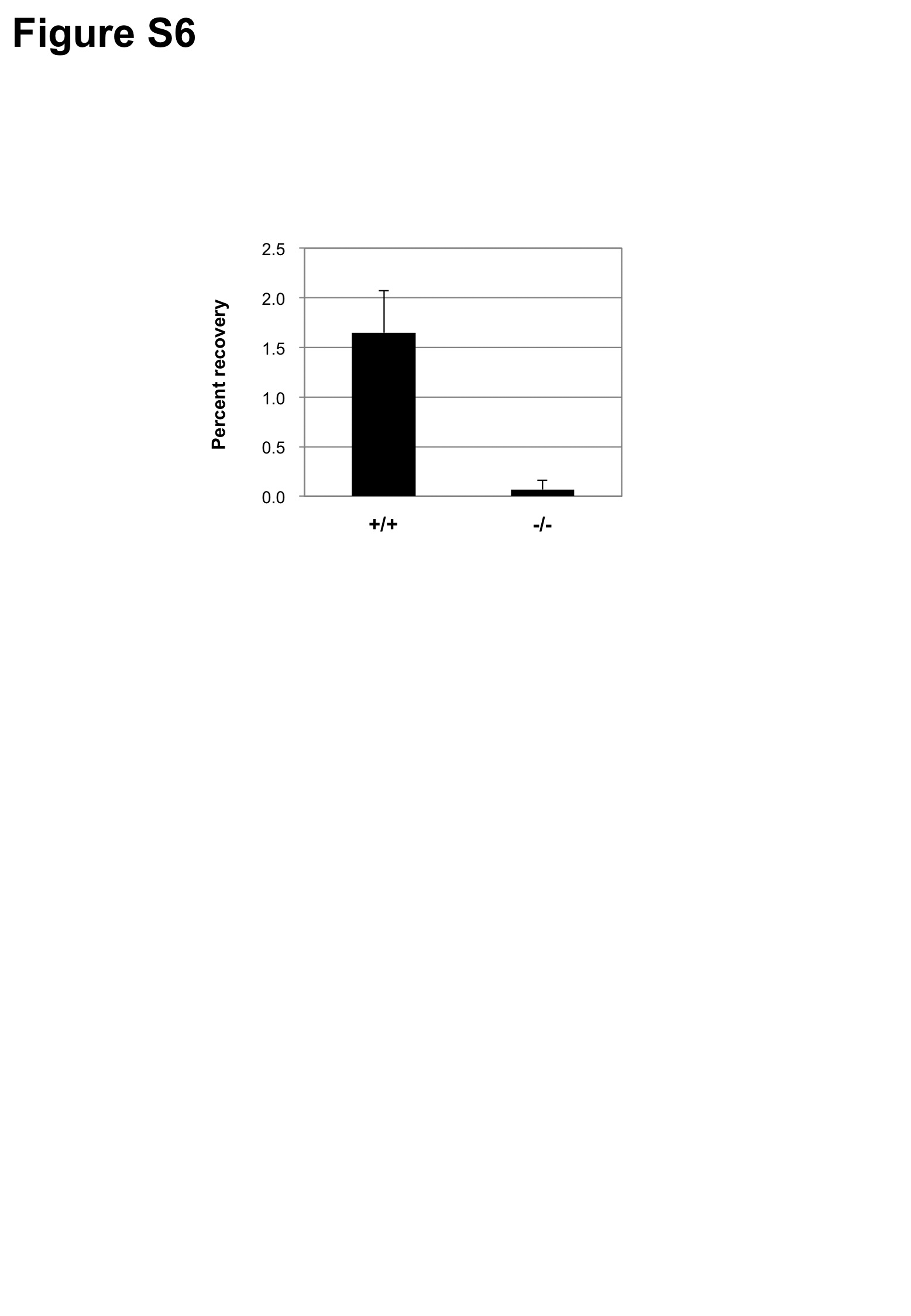

Supplement: Figure S6 — Bone marrow homing assay of Robo4-mutant cells. Immature hematopoietic cells (c-Kit+Lin- cells) were sorted from WT or Robo4−/− BM cells and were labeled with CSFE. Labeled cells were then transplanted into lethally irradiated recipient mice. 16 hours after transplantation, BM cells were harvested from recipient mice and analyzed for CSFE-positive cells by flow cytometry. The data are mean+/−S.D. (n = 3). Difference was statistically significant by Student’s t-test (p<0.05). (TIF) [file pone.0050849.s006.tif]
